# Prognostic role of DEK in human solid tumors: a meta-analysis

**DOI:** 10.18632/oncotarget.19684

**Published:** 2017-07-29

**Authors:** Gang Liu, Disheng Xiong, Junjie Zeng, Guoxing Xu, Rui Xiao, Borong Chen, Zhengjie Huang

**Affiliations:** ^1^ Department of Gastrointestinal Surgery, First Affiliated Hospital of Xiamen University and Xiamen Cancer Hospital, Xiamen, People's Republic of China; ^2^ Department of Gastrointestinal Surgery, First Clinical Medical College of Fujian Medical University, Fuzhou, People's Republic of China

**Keywords:** DEK, solid tumors, meta-analysis

## Abstract

Recently, the oncogenic role of DEK has been recognized in several cancer types. However, its prognostic role in human solid tumor remains unclear. Thus, the present meta-analysis, based on 14 published studies (2208 patients) searched from PubMed, Web of Science, and EMBASE databases, assessed the prognostic value of DEK in human solid tumors. Furthermore, the pooled hazard ratio (HR) for overall survival (OS) was evaluated with fixed-effects models. A subgroup analysis was also performed according to the patients’ ethnicities and tumor types. Data from these published studies were extracted, and the results showed that the overexpression of DEK was significantly associated with poor OS in human solid tumors. The combined hazards ratio was (HR = 1.83; 95% CI, 1.64–2.05, *P* < 0.00001) for OS (univariable analysis) with a fixed-effects model without any significant heterogeneity (*P* = 0.71, I^2^ = 0%). The combined HR was (HR = 1.70; 95% CI, 1.48–1.96, *P* < 0.00001) for OS (multivariable analysis) with a fixed-effects model, and no significant heterogeneity was observed (*P* = 0.36, I^2^ = 9%). Therefore, the overexpression of DEK was correlated with poor survival in human solid tumors, which suggests that the expression status of DEK is a valuable biomarker for the prediction of prognosis and serves as a novel therapeutic target in human solid tumors.

## INTRODUCTION

Human solid tumors are the most common malignant tumors and also the leading cause of cancer-related deaths in worldwide morbidity and mortality rate [1, 2]. Although the cancer-related studies have reported that numerous biomarkers are involved in solid tumors, only a few biomarkers have been clearly substantiated for clinical usage [3]. On the other hand, the lack of biomarkers for early diagnosis and weak prognostic significance of the histological indicators limited the efficiency of current treatment for malignant tumors and molecular markers for targeted therapy. Therefore, detecting the tumor-related markers involved in solid tumors is critical for the improvement of diagnosis, therapy, and prognosis prediction of cancer.

Recently, the oncogene *DEK* has become a topic of intensive research; it was originally identified as one of the parts of the *DEK-CAN* fusion gene, arising from the translocation (6;9) (p23;q34), identified in patients with a subtype of acute myeloid leukemia [4]. The gene encodes a 375 amino acid protein with a molecular weight of 43 kDa [5]. Some studies reported that *DEK* gene is involved in various fundamental nuclear processes. Kavanaugh et al. suggested that DEK expression modulates DNA-dependent protein kinase (DNA-PK) signaling and the efficiency of DNA double-strand break (DSB) repair [6]. The protein encoded by the proto-oncogene DEK changes the topology of chromatin and reduces the efficiency of DNA replication in a chromatin-specific manner [7]. McGarvey et al. has been identified the DEK as one of the first factors that associates with mRNA in a splicing-dependent manner, indicating that it could function to coordinate splicing with one or more subsequent steps in gene expression [8]. In other words, the functions of DEK involve DNA damage repair, DNA replication and mRNA splicing. Several studies also have reported that the *DEK* gene might accelerate tumorigenesis and neoplastic progression owing to interference with cell division, apoptosis, senescence, and inhibition of cell differentiation, as well as, cooperate closely with the transformation of oncogenes [6, 7, 9, 10].

A large number of studies suggested that the overexpression of DEK was associated with poor survival of patients with various types of solid tumors, including lung cancer [11–13], hepatocellular carcinoma [14–16], colorectal cancer [17–19], breast cancer [20–22]; gastric cancer [23, 24], prostate cancer [25], pancreatic cancer [26], melanoma [27–29], and ovarian tumors [30]. Accordingly, we performed an exhaustive meta-analysis to appraise the prognostic merit of DEK overexpression in human solid tumors. The aim of this meta-analysis was to assess the correlation of the high expression of DEK with survival in human solid tumors and illustrate the clinical value of DEK serving as a potential therapeutic target and prognostic indicator for human solid tumor patients.

## RESULTS

### Study selection and characteristics

**Figure 1:**
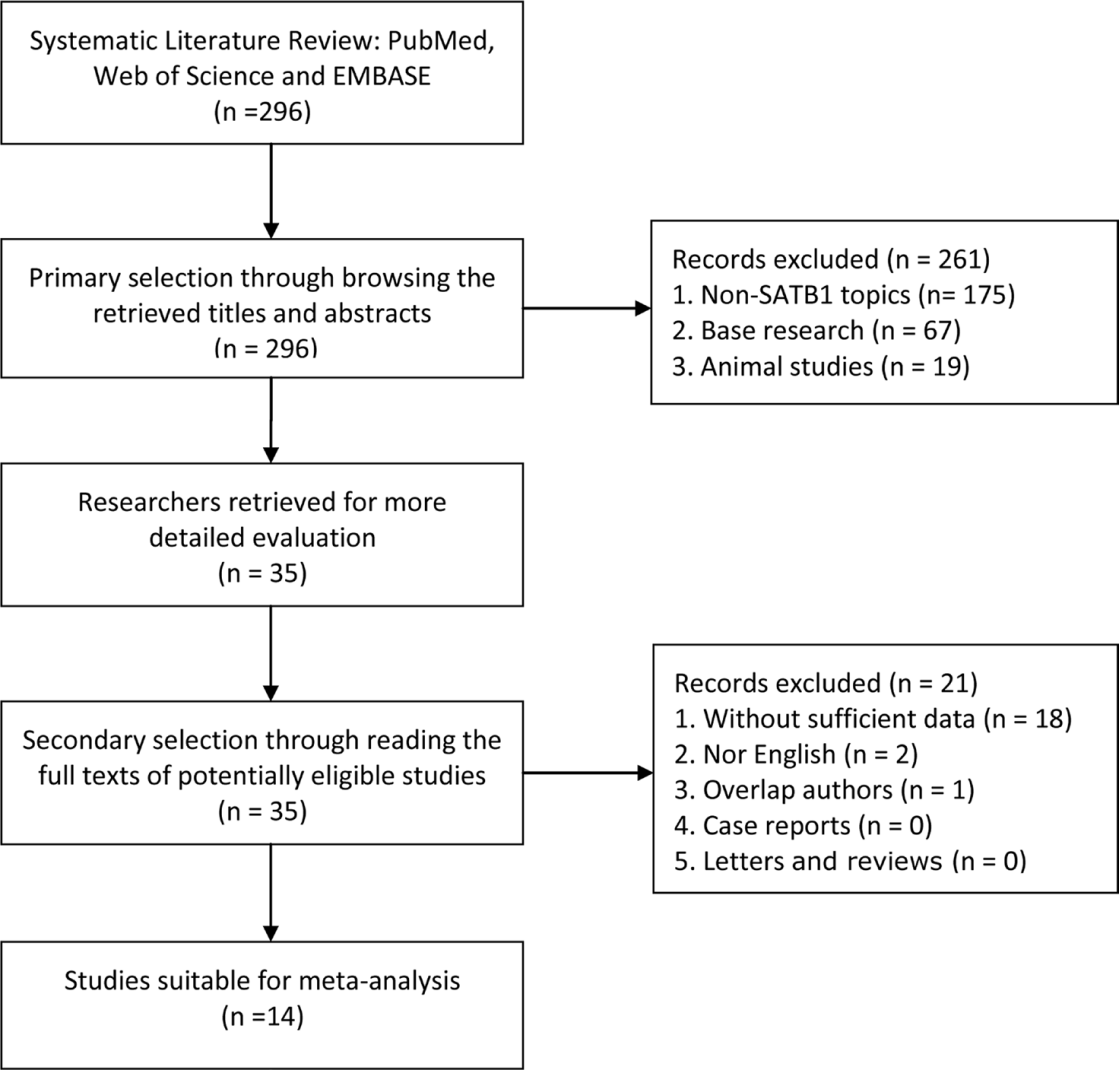
Flow diagram shows search strategy

296 relevant studies were retrieved initially. After using the search strategy described above, a total of14 studies comprising 2208 cases were considered in this meta-analysis (Figure 1). The major baseline characteristics of the 14 eligible publications were reported in Table 1. The search was conducted encompassing 4 countries (China, Japan, Spain, and Canada) on the literature that was published between 2011 and 2017. Among these, 13 studies reported OS with respect to lung cancer and hepatocellular carcinoma (*n* = 3), colorectal cancer and gastric cancer (*n* = 2), breast cancer, prostate cancer, pancreatic cancer, and melanoma (*n* = 1), and 1 study presented data for progression-free survival (PFS).

**Table 1:** The characteristics of the included studies

References	Country	Type of cancer	Patient No.	Male / Female	TNM staging	Detect method (Cut-off)	Increased DEK (%)	Fellow-up (months)	Survival analysis	HR (95%CI)	HR (Obtain)
Shibata T, et al. (2010)	Japan	LC	79	NR	NR	IHC (NR)	35 (44.3%)	120	OS (U)	1.87 (1.10–3.19)	Curve
Lin LJ, et al. (2013)	China	HCC	178	116/62	I–IV	IHC (> 5%)	86 (48.3%)	60	OS (U)	1.94 (1.45–2.60)	Direct
Lin L, et al. (2013)	China	CRC	99	87/22	I–III	IHC (>25%)	53 (53.5%)	60	OS (U) OS (M)	1.54 (1.06–2.25) 1.81 (1.21–2.71)	Direct
Lin D et al. (2014)	Canada	NEPC	160	NR	I–IV	IHC (Score > 1)	5 (3.1%)	120	DFS (U) DFS (M)	16.98 (3.59–80.38) 6.91 (1.33–35.96)	Direct
Martinez UJ, et al. (2014)	Spain	CRC	67	47/20	NR	IHC (NR)	21 (31.3%)	120	OS (U) OS (M)	2.83 (1.24–6.46) 2.41 (1.04–5.58)	Direct
Piao J, et al. (2014)	China	GC	172	102/70	I–IV	IHC (> 25%)	114 (70.3%)	84	OS (U) OS (M)	1.58 (1.15–2.17) 1.42 (1.03–1.96)	Direct
Wang X, et al. (2014)	China	LC	130	72/58	I–III	IHC (> 25%)	58 (44.6%)	60	OS (U) OS (M)	1.63 (1.15–2.31) 1.59 (1.09–2.33)	Direct
Yi HC, et al. (2015)	China	HCC	55	43/12	I–IV	IHC (> 25%)	26 (47.2)	60	OS (U) OS (M)	2.27 (1.47–3.51) 2.97 (1.73–5.10)	Direct
Ying G, et al. (2015)	China	BC	628	NR	I–III	IHC (NR)	389 (61.9%)	60	OS (U)	2.70 (1.49–4.88)	Curve
Ou Y, et al. (2016)	China	GC	192	148/44	I–IV	IHC (> 50%)	84 (43.8%)	60	OS (U) OS (M)	1.77 (1.16–2.71) 1.80 (1.09–2.97)	Direct
Riveiro FE, et al. (2016)	Spain	Melanoma	99	47/52	NR	IHC (> 25%)	42 (42.4%)	120	OS (U) OS (M)	2.28 (1.31–3.97) 2.03 (1.11–3.71)	Direct
Liu X, et al. (2016)	China	LC	196	109/87	I–IV	IHC (> 5%)	130 (66.3%)	96	OS (U) OS (M)	1.53 (1.13–2.07) 1.39 (1.02–1.89)	Direct
Yu L, et al. (2016)	China	HCC	66	NR	NR	PCR	33 (50%)	60	OS (U)	1.96 (1.24–3.10)	Curve
Sun J, et al. (2017)	China	PDAC	87	48/39	I–IV	IHC (> 50%)	46 (52.9)	30	OS (U) OS (M)	2.28 (1.45–3.58) 2.02 (1.29–3.16)	Direct

BC: Breast Cancer; CI: Confidence interval; CRC: Colorectal Cancer; DFS: Disease-free survival; GC: Gastric cancer; HCC: Hepatocellular Carcinoma; HR: Hazard ratio; IHC: Immunohistochemistry; LC: Lung Cancer; M: Multivariable analysis; NEPC: Neuroendocrine prostate cancer; NR: Not Reported; OS: Overall survival; PDAC: Pancreatic ductal adenocarcinoma; U:Univariable analysis.

### Overall survival

**Figure 2:**
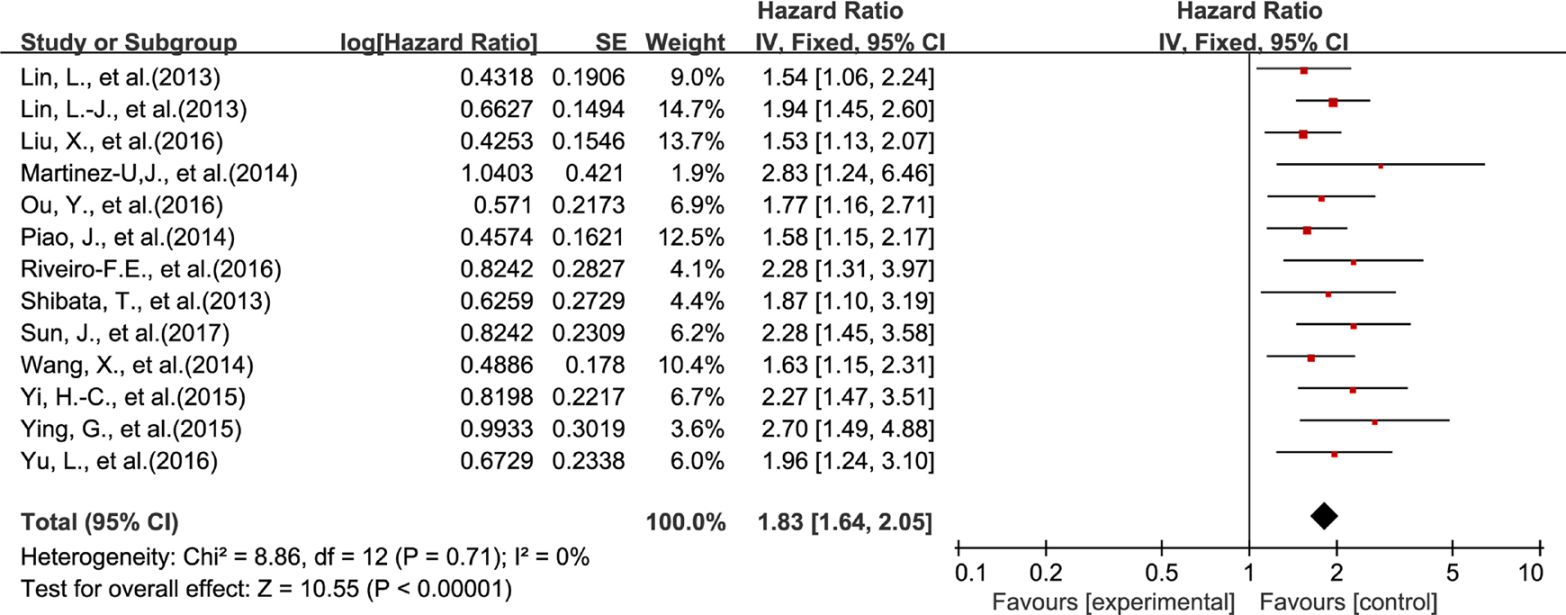
Meta-analysis of the association between DEK and OS (univariable analysis)

**Figure 3:**
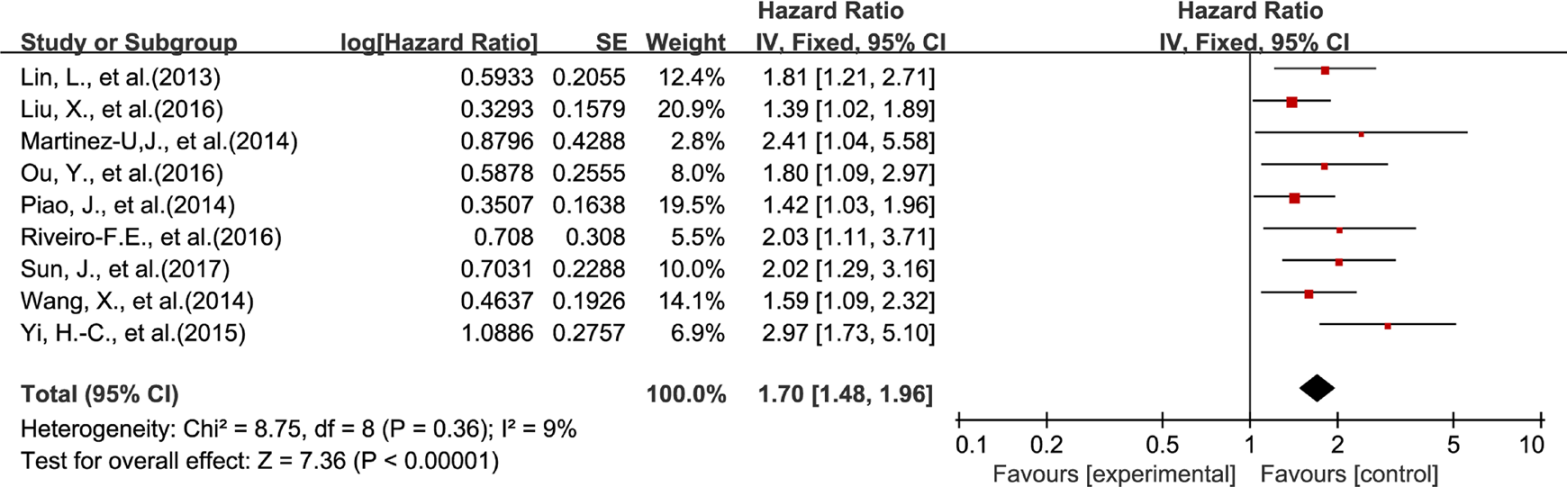
Meta-analysis of the association between DEK and OS (multivariable analysis)

The association between DEK and prognosis was shown in Figure 2 and Figure 3. The results demonstrated that DEK overexpression was associated with poor survival outcome of solid tumor patients. The combined HR (HR = 1.83; 95% CI, 1.64–2.05, *P* < 0.00001) for OS (univariable analysis) with a fixed-effects model showed no significant heterogeneity among the 13 included studies (*P* = 0.71, I^2^ = 0%) (Figure 2). The combined HR (HR = 1.70; 95% CI, 1.48–1.96, *P* < 0.00001) for OS by multivariable analysis with a fixed-effects model also showed no significant heterogeneity among the 9 included studies (*P* = 0.36, I^2^ = 9%) (Figure 3).

To explore the heterogeneity, the subgroup analyses for OS were performed based on the types of tumor and ethnicity of the patients. The results (Table 2) showed that HR (95% CI) for OS (univariable and multivariable analysis) in the digestive system neoplasms and other types of tumors was 1.87 (1.62–3.10), 1.83 (1.52–2.19), 1.77 (1.47–2.13), and 1.53 (1.23–1.97), respectively. In addition, the results showed that HR (95% CI) for OS (univariable and multivariable analysis) in Asian and Caucasian was 1.80 (1.6–02.02), 1.67 (1.44–1.93), 2.24 (1.54–3.86), and 2.15 (1.32–3.51), respectively. As displayed in Table 3, the quality assessment of all the eligible studies was performed by the modified Newcastle–Ottawa Scale (NOS). The median quality score of these studies was 8, indicating that the quality of the methodology was relatively high.

**Table 2:** Pooled HR for OS according to subgroup analysis

References	No. of patients	No. of studies	Fixed-effect model		Heterogeneity	
			HR (95% CI)	*P* value	I^2^ (%)	*P* value
**Analysis type**						
**Univariate**	2048	13	1.83 (1.64–2.05)	< 0.00001	0%	0.71
**Multivariate**	1097	9	1.70 (1.48–1.96)	< 0.00001	9%	0.36
**Tumor type** (Univariate)						
**Digestive system neoplasm**	916	8	1.87 (1.62–2.15)	< 0.00001	0%	0.69
**Others**	1096	5	1.77 (1.47–2.13)	< 0.00001	0%	0.42
**Tumor type** (Multivariate)						
**Digestive system neoplasm**	672	6	1.83 (1.52–2.19)	< 0.00001	18%	0.30
**Others**	389	3	1.53 (1.23–1.97)	< 0.00001	0%	0.53
**Ethnicity** (Univariate)						
**Asian**	1704	11	1.80 (1.60–2.02)	< 0.00001	0%	0.72
**Caucasian**	166	2	2.24 (1.54–3.86)	< 0.00001	0%	0.67
**Ethnicity** (Multivariate)						
**Asian**	931	7	1.67 (1.44–1.93)	< 0.00001	22%	0.26
**Caucasian**	166	2	2.15 (1.32–3.51)	< 0.00001	0%	0.75

**Table 3:** Quality assessment of eligible studies with Newcastle-Ottawa Scale

References	Year	Selection	Comparability	Outcome	NOS
Shibata T, et al.	2010	⋆⋆⋆	⋆	⋆⋆	6
Lin LJ, et al.	2013	⋆⋆⋆	⋆⋆	⋆⋆	7
Lin L, et al.	2013	⋆⋆⋆	⋆⋆	⋆⋆⋆	8
Lin D, et al.	2014	⋆⋆⋆	⋆	⋆⋆	6
Martinez UJ, et al.	2014	⋆⋆⋆	⋆⋆	⋆⋆	7
Piao J, et al.	2014	⋆⋆⋆	⋆⋆	⋆⋆⋆	8
Wang X, et al.	2014	⋆⋆⋆	⋆⋆	⋆⋆⋆	8
Yi HC, et al.	2015	⋆⋆⋆	⋆⋆	⋆⋆⋆	8
Ying G, et al.	2015	⋆⋆⋆	⋆	⋆⋆	6
Ou Y, et al.	2016	⋆⋆⋆	⋆⋆	⋆⋆⋆	8
Riveiro FE, et al.	2016	⋆⋆⋆	⋆⋆	⋆⋆⋆	8
Liu X, et al.	2016	⋆⋆⋆	⋆⋆	⋆⋆⋆	8
Yu L, et al.	2016	⋆⋆⋆	⋆	⋆⋆	6
Sun J, et al.	2017	⋆⋆⋆	⋆⋆	⋆⋆⋆	8

### Publication bias

**Figure 4:**
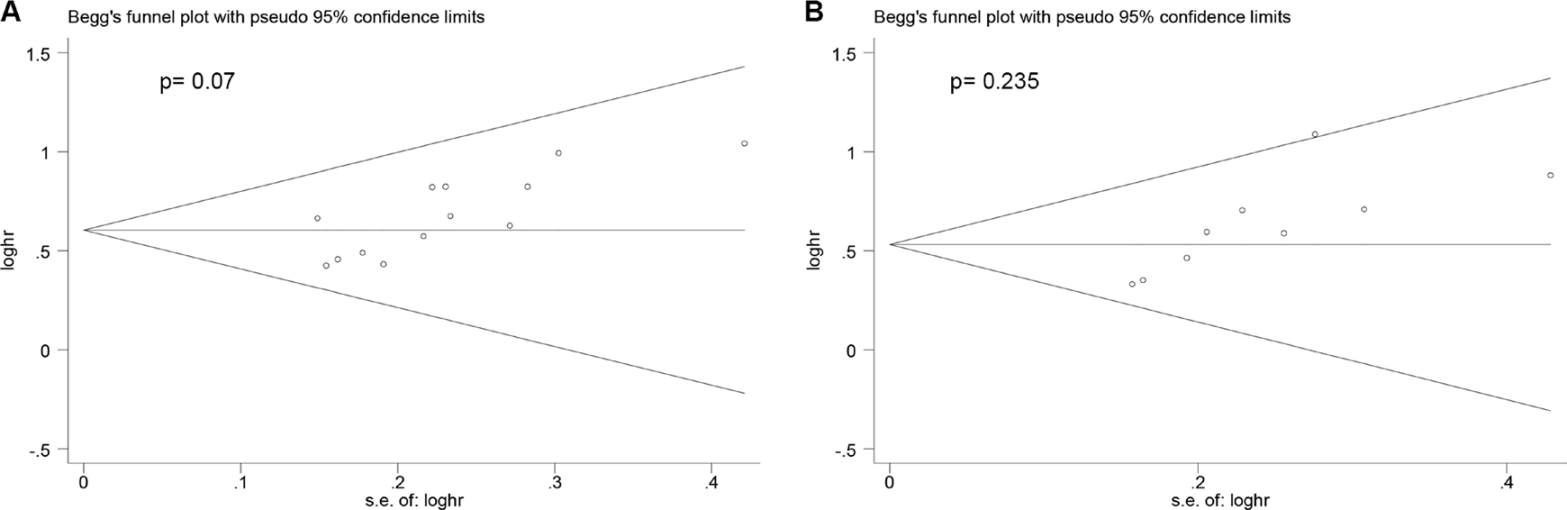
Summary of Begg's funnel plots of publication bias for OS in all patients (**A**) univariable analysis; (**B**) multivariable analysis.

Begg's funnel plot and Begg's test were conducted to assess the publication bias of the included trials. As is shown in Figure 4, the shape of the funnel plots presented no significant asymmetry. The *P* values of Begger's test for OS with univariable and multivariable analysis were 0.07 and 0.235, respectively, indicating the absence of an obvious publication bias in these analyses.

## DISCUSSION

In order to obtain the prognostic significance of a potential biomarker, over the past decades, researchers have been dedicated to identifying the new prognostic markers for better clinical decisions regarding therapy and outcomes. The prognostic value of DEK expression has been investigated extensively in human solid tumor, including lung cancer [11–13], hepatocellular carcinoma [14–16], colorectal cancer [17–19], breast cancer [20–22], gastric cancer [23, 24], prostate cancer [25], pancreatic cancer [26], melanoma [27–29], and ovarian tumors [30]. Therefore, it is essential to summarize the prognostic value of DEK in solid tumors. Therefore, by summarizing the results of the published studies, we aimed to evaluate the correlations between DEK and tumors and provide valuable information for clinical decision-making in solid tumors.

As a well-established oncogene, DEK was associated with not only chromatin reconstruction and gene transcription, but also important in proliferation of cells and cell apoptosis [22, 30–33]. Han et al. revealed that the DEK was closely involved in the proliferation of serous ovarian cancer cells and that the high level of DEK expression was significantly associated with the increased proliferating index of Ki-67 [30]. Privette et al. demonstrated that DEK oncogene promotes cellular proliferation through paracrine Wnt signaling in Ron receptor-positive breast cancers [22]. Wise-Draper et al. reported that apoptosis inhibition by the human DEK oncoprotein involves interference with p53 functions [33]. These findings indicate that DEK plays important roles in the progression of tumor cells.

Accumulating evidence has suggested that DEK overexpression is associated with not only the development and progression of solid tumors, but also with an unfavorable prognosis. Sun et al. reported that DEK overexpression was an independent prognostic factor along with histological grade and TNM stage, and also influenced OS rates of pancreatic cancer in grade 1 and 2, and early-stage groups [26]. Non-small cell lung carcinoma (NSCLC) patients with DEK expression had lower disease-free and overall survival rates compared with those without DEK expression [11–13]. However, Liu et al. showed that DEK expression status was not related to the survival of NSCLC patients with an advanced clinical stage [13]. Some studies revealed that upregulation of DEK in patients with gastric cancer was associated with the presence of large tumors, serosal invasion, a poorer tumor grade, lymph node metastasis and increased stage tumors [23, 24]. Therefore, DEK might be a potential prognostic factor to predict the survival in patients with solid tumors.

To our knowledge, this is the first systematic review elaborate about the association of DEK overexpression with OS of human solid tumors. We systematically evaluated the survival data of 2208 patients with solid tumors included in the 14 eligible studies. The results suggested that the overexpression of DEK is a biomarker of poor prognosis in human solid tumors, with similar results of OS for different types of tumors; the high level of DEK expression was related to poor OS among different types of solid tumors. Thus, further studies are imperative to clarify the underlying mechanism and role of DEK in pathogenesis and prognostic merit in human solid tumors.

The present meta-analysis involved several pivotal implications. First, it revealed that DEK expression was correlated with unfavorable outcomes in different types of solid tumors, thereby suggesting DEK as a novel therapeutic target. Finally, the study emphasizes the potential clinical application of DEK as a valuable prognostic biomarker. Nevertheless, this meta-analysis also has some limitations. First, some research results might not be published, which inevitably leads to publication bias. Second, the different methods of analysis and the cut-off values for evaluating DEK overexpression are inconsistent. Finally, since some HR could not be extracted directly from the articles, we calculated the HR according to the Kaplan–Meier survival curves, which renders less reliability to the results. In summary, our meta-analysis indicates that DEK overexpression is associated with poor OS in all the human solid tumors, suggesting it to be a valuable prognostic indicator and a novel therapeutic target for human solid tumors.

## MATERIALS AND METHODS

### Search strategy

We conducted an exhaustive search of PubMed, Web of Science, and EMBASE for studies measuring the expression of DEK and survival in patients with solid tumors until January 2017. The search included the terms “DEK” and “neoplasms” or “cancer” or “tumor” or “survival” or “prognosis”, and the results were restricted to human studies of solid tumors. All the potential studies were carefully searched for in the references.

### Inclusion and exclusion criteria

The inclusion criteria were as follows: (1) Investigated the association between DEK and patients’ prognosis [(OS and/or disease-free survival (DFS)]; (2) A follow-up period no less than 30 months; (3) Only English language studies were included; (4) When the same author reported results from the same patient population, the most recent report or a complete one was included. The exclusion criteria were as follows: (1) Studies of animal experiments, or no human solid tumors; (2) Studies without sufficient data for obtaining HR and 95% CI; (3) Duplicate reports and inappropriate article types, such as case reports, letters, conference papers, and reviews; (4) Published in a language other than English. All the eligible manuscripts were carefully scrutinized by two independent authors. To reach a consensus, disagreements on the conflicting results were resolved between the two authors.

### Data extraction and quality assessment

The cohort studies, included in this meta-analysis, were assessed by two independent authors with respect to references, country, type of cancer, number of patients, gender of patients, detection method, cut-off value (%), increased DEK (%), duration of follow-up, survival analysis, HR (95% CI), HR (obtain), and NOS scores. In this meta-analysis, the OS data were extracted from the tables or Kaplan–Meier curves for both DEK negative and positive groups. To identify the high-quality studies, each publication was scored based on the NOS [34]. This scale varies from 0–9 stars; studies with a score ≥ 6 were considered methodologically adequate. A consensus NOS score for each item was achieved by discussion.

### Statistical analysis

HR (95% CI) was used to evaluate the prognostic value of DEK overexpression in human solid tumors. Normally, the statistical variables were extracted directly from the primary studies, or calculated using the Kaplan–Meier method according to Tierney's method [35]. Higgin's I^2^ statistics and Cochran's *Q* tests calculated the heterogeneity of the individual HR [36, 37]. A probability value of *P* < 0.05 and/or I^2^ > 50% indicated significant heterogeneity, and a random-effects model was used depending on the heterogeneity analysis. Otherwise, a fixed-effect model was applied. Subgroup analysis was further conducted for interpretation of identified heterogeneity. The publication bias was estimated using the funnel plots with Begg's test, and results with *p* values of less than 0.05 were considered to be indicative of significant bias. Stata 12.0 software and Review Manager version 5.3 were used for all statistical analyses in this meta-analysis.
